# Supplementary figures and images for: In depth analysis of patients with severe SARS-CoV-2 in sub-Saharan Africa demonstrates distinct clinical and immunological profiles
Source: medRxiv. 2021 Feb 20:2021.02.15.21251753. Preprint. [Version 2] doi: 10.1101/2021.02.15.21251753 (PMC7899472; doi:10.1101/2021.02.15.21251753)

Figure S1

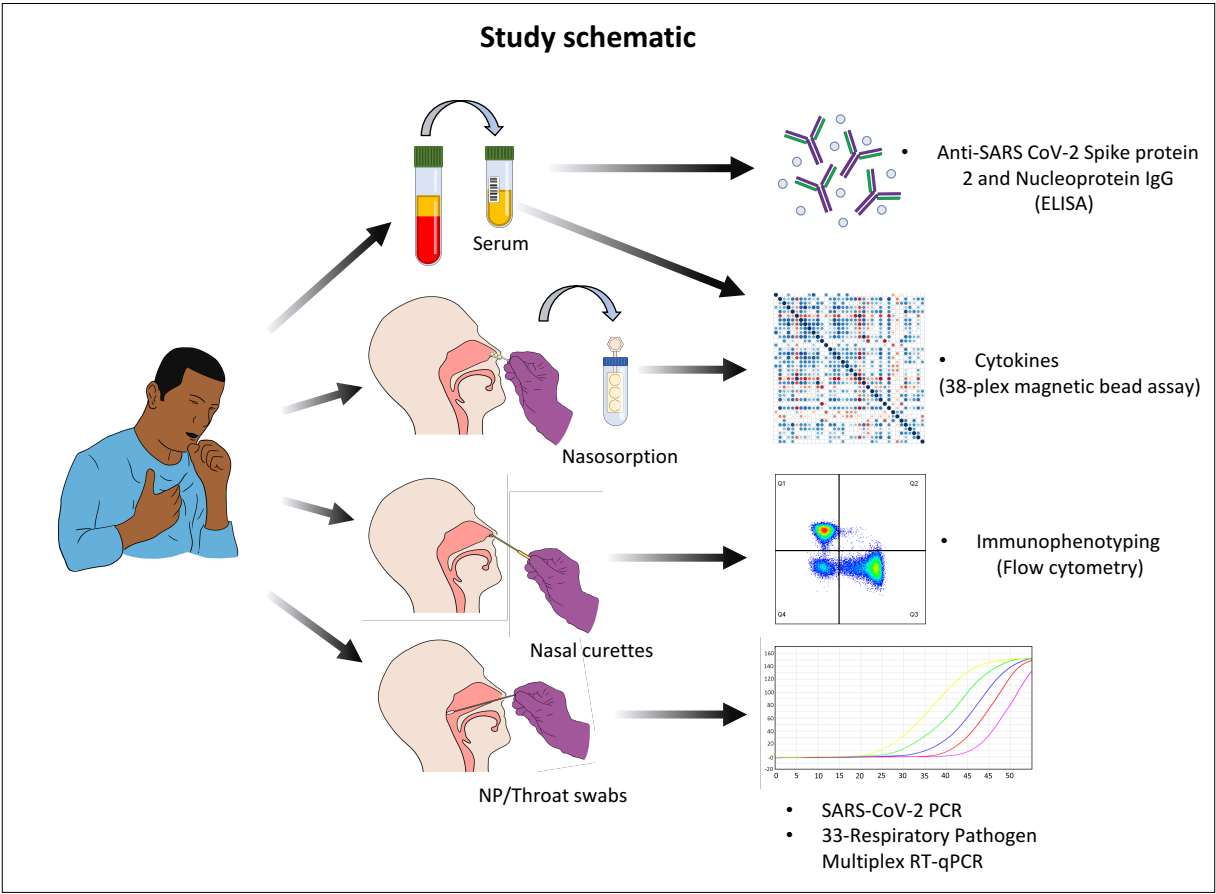

Figure S2

Nasal lining fluid

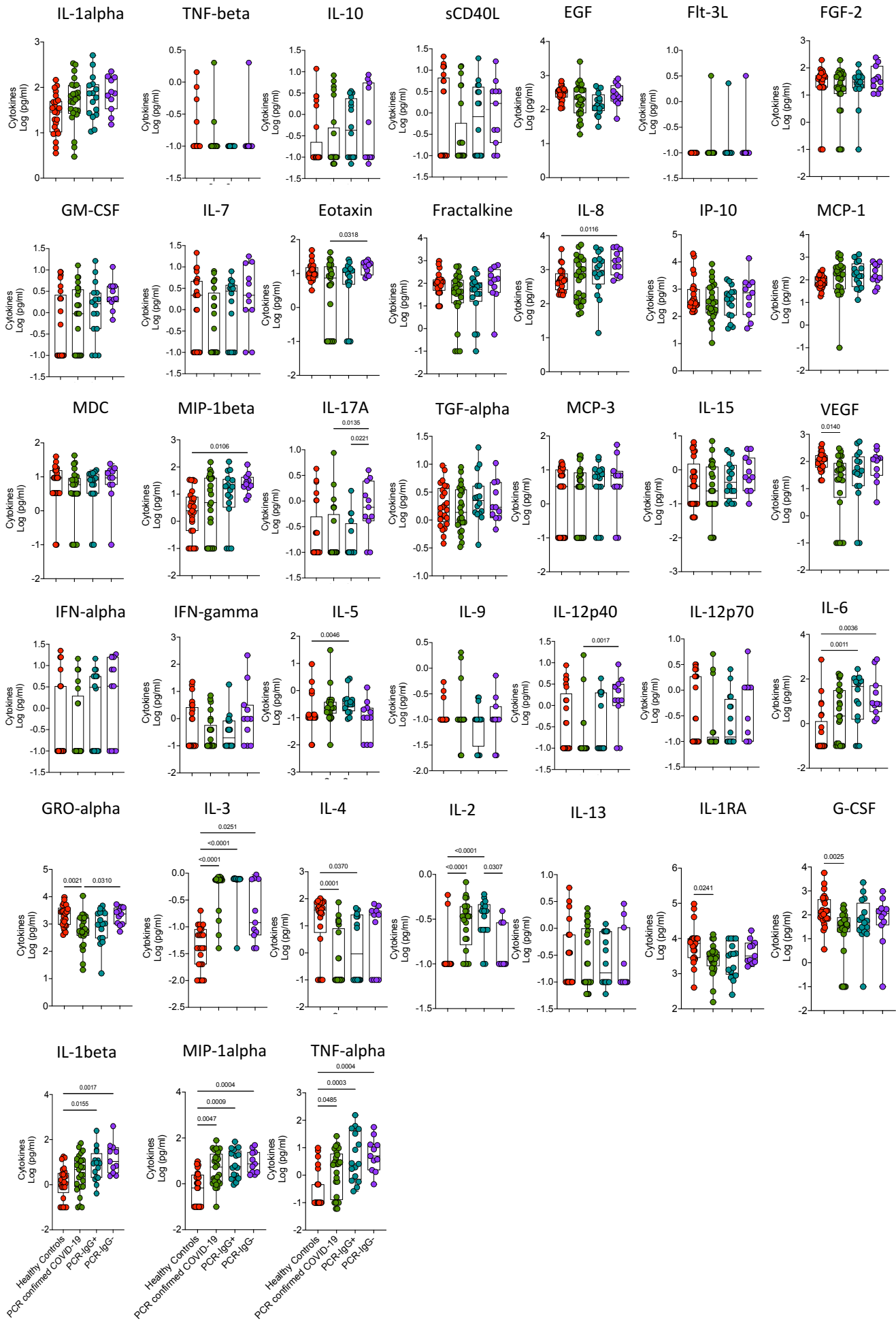

Figure S3

Serum

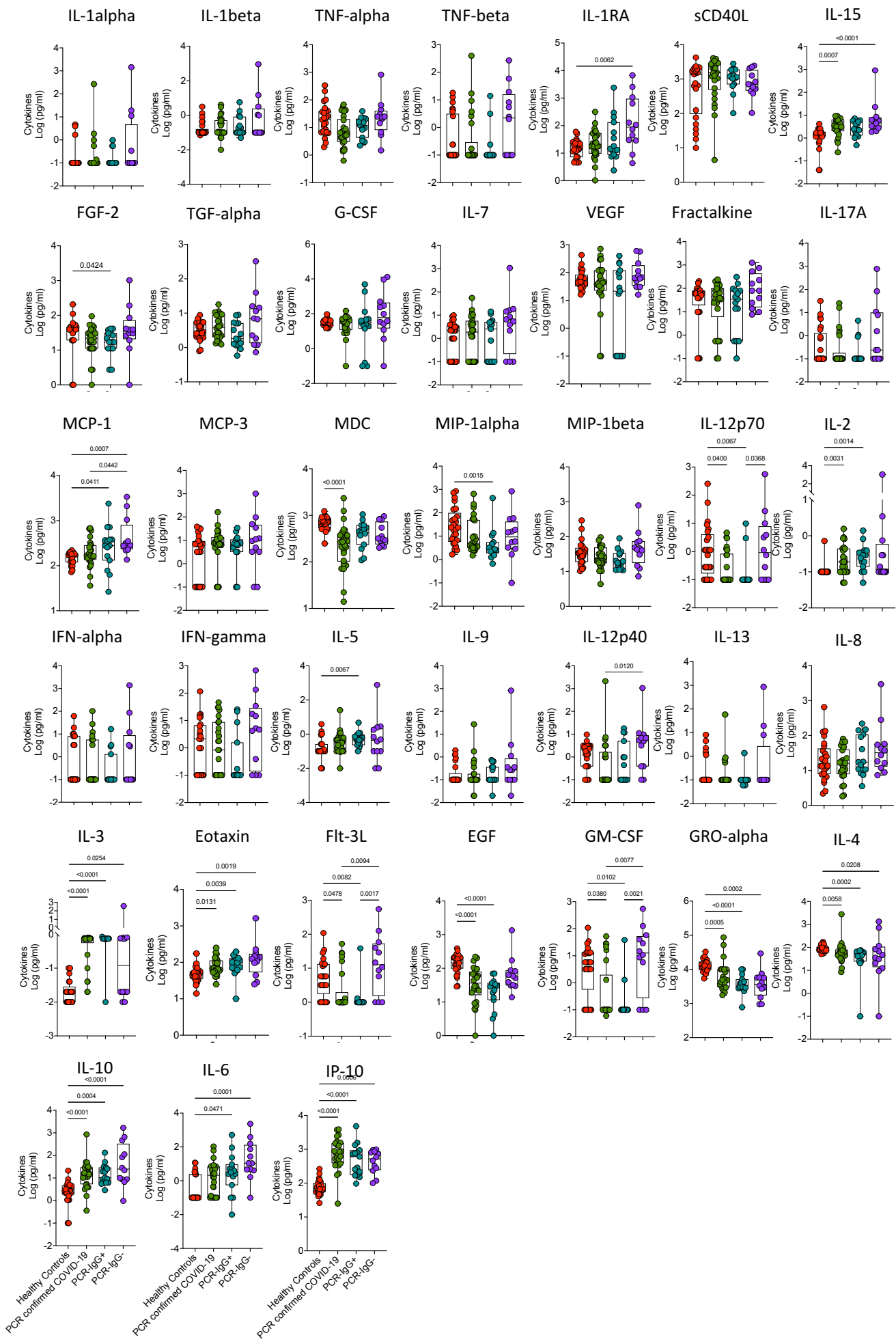

Figure S4

a.

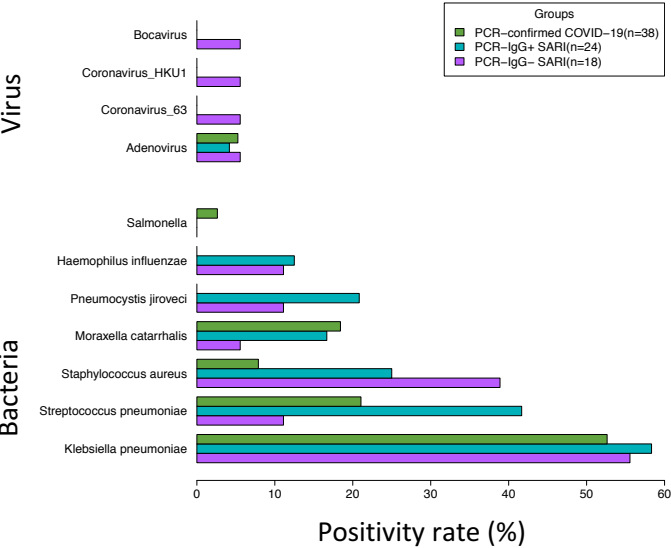

b.

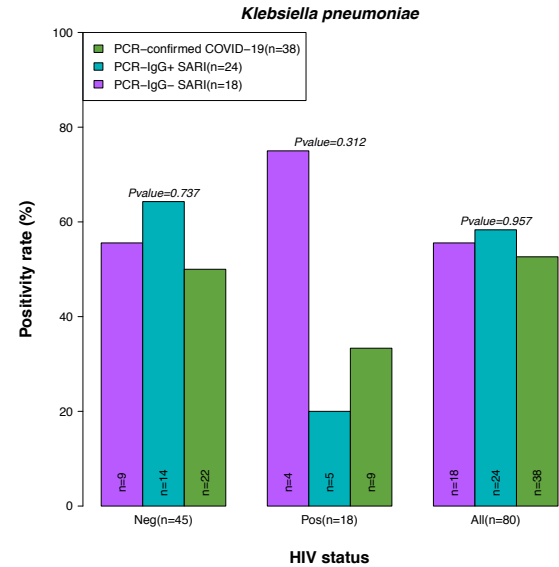

Supplement: Supplement 1 [file media-1.pdf]
